# The effect of Sequential Retraction of Maxillary Incisors on Anterior Gingival Biotypes

**DOI:** 10.12669/pjms.42.9.13330

**Published:** 2026-09

**Authors:** Muhammad Ihtisham Munawar, Ambreen Afzal Ehsan, Hasnain Sakrani, Sadaf Siddiqui

**Affiliations:** 1 Muhammad Ihtisham Munawar, Department of Orthodontics, Altamash Institute of Dental Medicine Karachi, Pakistan; 2 Ambreen Afzal Ehsan, Department of Orthodontics, Altamash Institute of Dental Medicine Karachi, Pakistan; 3 Hasnain Sakrani, Department of Orthodontics, Altamash Institute of Dental Medicine Karachi, Pakistan; 4 Sadaf Siddiqui, Department of Orthodontics, Altamash Institute of Dental Medicine Karachi, Pakistan

**Keywords:** Angle Class-II, Gingival biotype, Gingival thickness, Probing depth, Sequential retraction

## Abstract

**Objective:**

To evaluate the effect of sequential retraction of maxillary incisors on the gingival biotypes

**Methodology:**

This cross-sectional was conducted in the Department of Orthodontics at Altamash Institute of Dental Medicine, Karachi. Duration of study was one year from June 2023 to July 2024. A total of 50 patients aged 18-30 years, diagnosed with Angle Class II Division 1 malocclusion and ANB > 4°, were included. Participants were categorized into two groups, thin (n=35) and thick (n=15) gingival biotypes. Gingival parameters were assessed before and after maxillary incisor retraction using a World Health Organization (WHO) probe also known as the Community Periodontal Index of Treatment Needs (CPITN) probe. Quantitative data, the gingival height, probing depth, and gingival width, were assessed using the Shapiro-Wilk test. Statistical analysis was performed using SPSS with level of significance set at p ≤ 0.05.

**Results:**

Average age of patients was 24.5 years, comprising 40% males and 60% females. Among them, 70% had a thin gingival biotype, and 30% had a thick biotype. Thin biotype patients had lower mean gingival thickness (0.88 mm) and width (3.2 mm) than thick biotype patients (1.25 mm and 3.5 mm, respectively). Probing depth and gingival recession were slightly higher in thin biotypes. THIn the thin biotype group, gingival thickness decreased by 0.06 mm, probing depth increased by 0.2 mm, gingival width reduced by 0.2 mm, and gingival recession rose by 0.2 mm. For the thick biotype, changes were less pronounced, with a decrease in gingival thickness by 0.03 mm, a slight increase in probing depth by 0.1 mm, a reduction in gingival width by 0.1 mm, and an increase in gingival recession by 0.1 mm. After sequential retraction of maxillary teeth patients with a thin biotype showed significant reductions in gingival thickness (p=0.03) and width (p=0.02) with an increase in probing depth (p=0.04) and gingival recession (p=0.01). In contrast, the thick biotype group demonstrated minimal and statistically insignificant changes in these parameters (p > 0.05).

**Conclusion:**

Patients with a thin biotype have increased risk of periodontal recession and reduction of gingival thickness while retraction of anterior teeth. Orthodontic treatments should consider appropriate measures to retract teeth keeping the biotype of gingiva in mind to preserve gingival health.

## INTRODUCTION

The major complaint of orthodontic patients is forward positioned teeth. One of the methods to deal with this problem is extraction of first premolars and retracting anterior teeth in that space.^1^ The retraction of maxillary incisors is a common procedure, particularly in cases where a protrusive dental profile needs to be managed especially in treatment of Class-II where maxilla or maxillary teeth are protrusive.^2^ For mild retraction cases, molar distalization can be a choice, but for moderate to severe ones, space must be created by doing bimaxillary premolar extraction followed by retraction of anterior teeth.^3^

Retraction of maxillary incisors in class II Cases can be done by retracting teeth En-masse En masse i.e six anterior teeth together with mini screws or loop mechanics or sequential retraction i.e retracting canine first then the anterior four incisors with elastomeric chains, coil springs or loop mechanics.^4^ Space closure can be achieved either by retracting En masse which is retraction in a single-step process encompassing anterior teeth including canines that moves together in extraction space while sequential retraction is a two-step process in which canine is retracted first followed by the central and lateral incisors.^5^

In clinical practice, an Orthodontist always looks for the methods and biomechanics that provide less treatment time and desirable anchorage control during space closure. The use of any of these methods highly depends on the choice of the clinician, the appliance system used, and the best treatment plan decided for the patient. Healthy gingiva and periodontium play an important role in retracting anterior teeth. The periodontium and its appearance clinically differ from individual to individual including the genotype, width of keratinized gingiva (WKG) and probing depth.^6^

The thickness of the gingival tissue in facio palatal aspect is described either thick or thin biotype. Thin gingival phenotype is delicate, remarkably scalloped, and is more likely to have plaque buildup, bleeding and gingival recession whereas subjects with thick biotypes have more extensive and less scalloped margins. The gingival biotype has a critical role on the outcome of the treatment.^7^ So it’s important to have an accurate diagnosis and special attention before orthodontic treatment to achieve desirable outcomes.^8^ Among these concerns, the health and stability of the maxillary anterior gingiva warrant special attention, as adverse effects on gingival tissues can lead to gingival recession, attachment loss, and compromised esthetics.^9^

A study conducted in Thailand on En masse retraction of maxillary anterior teeth showed the gingiva gained more thickness (T_2_ = 0.90 ± 0.16) and length on the labial than palatal sides (T_2_ = 2.61 ± 0.51). The height loss occurred more in thin (T_2_ = 1.77 ± 0.17) than thick gingival biotype (T_2_ = 2.05 ± 0.44).^4^ A study conducted in Navi Mumbai on the gingival width, gingival thickness, papillary fill and probing depth in maxillary anterior teeth showed that the mean gingival width in thick and thin gingival phenotype was 4.2284mm and 4.6964mm respectively.^6^ To the best of our knowledge there are no previous studies on maxillary incisors retraction and its effect on gingiva in different gingival biotypes by using sequential retraction biomechanics. Orthodontic treatment objectives often focus on optimizing esthetics and functionality by correcting malocclusions and enhancing smile aesthetics. Understanding these biotypes is essential for clinicians to anticipate and manage the periodontal response during orthodontic treatment effectively.^10^ The objective of this study was to determine changes in gingival parameters in the anterior maxillary region of patient’s having thick and thin biotypes, by using sequential retraction method of retracting anterior teeth.

## METHODOLOGY

The study was conducted in the General Outpatient Department (OPD) at the Department of Orthodontics, Altamash Institute of Dental Medicine, Karachi. Duration of study was one year from June 2023 to July 2024.The objective was to assess effects of sequential retraction of maxillary incisors on the maxillary anterior gingiva across different gingival biotypes. Non-probability consecutive sampling was performed to include participants who met the specified inclusion criteria until the required sample size was achieved. The sample size was determined using the WHO calculator, with gingival thickness after sequential retraction set at 0.90 ± 0.16 (used as a reference parameter)4^4^ with a margin error of 5%. The reference parameter represents the mean and standard deviation of soft tissue thickness post retraction which was set as a standard for measuring anterior gingiva of maxillary region. The minimum calculated sample size was 40. Data was collected by one operator independently which was verified by second operator to ensure accuracy. Two operators collected the data. Prior to study both operators underwent training by periodontology department to ensure consistency and reliability Probing depth was measured using probe transparency method by WHO periodontal probe at six sites around the maxillary incisors to ensure an accurate representation of gingival health. Gingival Width was measured alongside other gingival parameters before and after retraction to observe any change.^11^

However, to ensure sufficient representation, a minimum of 50 cases were included in the study to safeguard against potential dropouts. There was a predominance of thick gingival biotype in the study sample attributed to the underlying distribution of gingival phenotypes and specific demographic characteristics of patients being recruited. Adult males and females, aged 18-30 years, with an Angle Class II Division one malocclusion with maxillary anterior teeth protrusion and an ANB angle > 4°, as measured on a lateral cephalometric radiograph. Patients who had undergone bilateral extraction of maxillary first premolars. Plaque and Gingival Index scores of one or less, based on the Sillness and Loe Classification.^6^ Gingival recession of 1-mm or less, according to Miller’s Classification.^12^ Probing depth of up to 3mm, measured with a WHO periodontal probe. No significant systemic diseases affect bone metabolism. No periodontal diseases. No history of trauma to the maxillary anterior teeth. The following patients were excluded from the study. Pregnant ladies, Presence of cleft lip or palate patients with history of previous orthodontic treatment or previous maxillary or periodontal surgery, current or past use of drugs associated with increased risk of gingival enlargement.

### Ethical Approval:

The study commenced after obtaining approval from the Ethics Review Committee (ERC) AIDM with the ERC No. AIDM/ERC/06/2023/01.Gingival parameters were assessed before and after the complete retraction of the maxillary incisors. The following clinical measurements were recorded for each participant:

**Table-I:** Demographic data of patients.

Variable	Total (n = 50)	Thin Biotype (n = 35)	Thick Biotype (n = 15)
Age (years)	24.5 ± 3.2	24.3 ± 3.1	24.9 ± 3.4
***Gender***			
- Male	20 (40%)	12 (34%)	8 (53%)
- Female	30 (60%)	23 (66%)	7 (47%)
ANB Angle (°)	5.5 ± 0.8	5.4 ± 0.7	5.6 ± 0.9
Plaque Index	0.8 ± 0.2	0.8 ± 0.2	0.7 ± 0.1
Gingival Index	0.9 ± 0.2	0.9 ± 0.2	0.8 ± 0.1
Gingival Thickness (mm)	1.0 ± 0.3	0.88 ± 0.17	1.25 ± 0.19
Probing Depth (mm)	2.0 ± 0.4	2.1 ± 0.5	2.0 ± 0.4
Gingival Width (mm)	3.4 ± 0.5	3.2 ± 0.6	3.5 ± 0.5
Gingival Recession (mm)	0.5 ± 0.1	0.5	0.4

### Gingival Thickness and Biotype Assessment:

The probe transparency method was used, where the visibility of the probe through the gingival tissue determined the biotype.

### Probing Depth and Gingival Height:

Probing depth was measured using a WHO periodontal probe at six sites around the maxillary incisors to ensure an accurate representation of gingival health.

### Gingival Width:

Gingival width which is the distance between the gingival margin(the edge of gum where it touches the tooth) and the mucogingival junction (border where the pink attached gingiva meets the mobile alveolar mucosa) was measured alongside other gingival parameters before and after retraction to observe any change.

Data was analyzed using SPSS software. Variables such as gender and gingival biotype were presented as frequencies and percentage distribution. Quantitative data, including gingival height, probing depth, and gingival width, were assessed using the Shapiro-Wilk test. For normally distributed data, results were presented in terms of mean ± standard deviation (SD).

**Fig.1:**
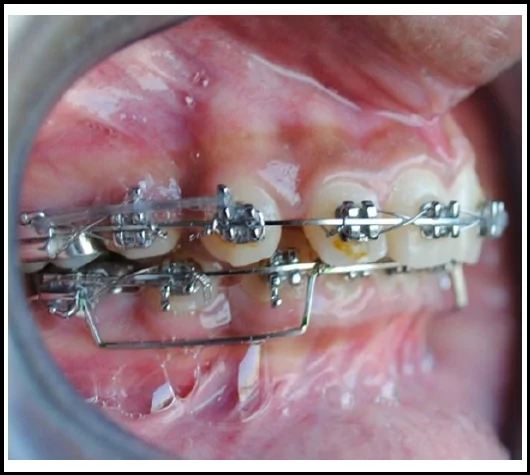
Sequential retraction: retracting canine 1st then all anterior teeth.

**Fig.2:**
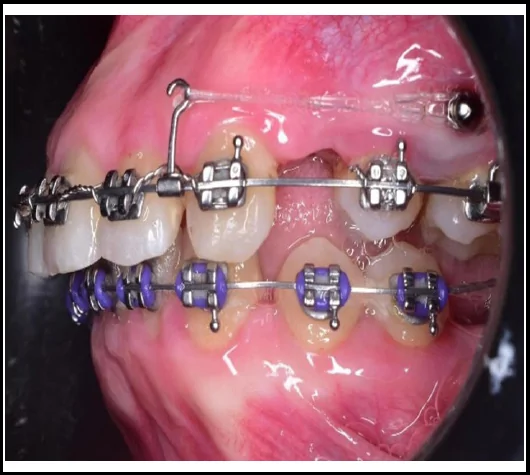
Enmasse retraction: retraction of all anterior teeth together.

**Table-II:** Baseline Gingival Biotype Characteristics by Gender.

Variable	Total (n = 50)	Male (n = 20)	Female (n = 30)	p-value
Age (years)	24.5 ± 3.2	24.7 ± 3.1	24.4 ± 3.3	0.42
ANB Angle (°)	5.5 ± 0.8	5.6 ± 0.7	5.4 ± 0.9	0.35
Plaque Index	0.8 ± 0.2	0.8 ± 0.2	0.8 ± 0.2	0.52
Gingival Thickness (mm)	1.0 ± 0.3	1.05 ± 0.28	0.95 ± 0.31	0.31
Probing Depth (mm)	2.0 ± 0.4	2.1 ± 0.5	2.0 ± 0.4	0.48
Gingival Width (mm)	3.4 ± 0.5	3.3 ± 0.5	3.4 ± 0.5	0.29
Gingival Recession (mm)	0.5 ± 0.1	0.5	0.5	0.37

## RESULTS

Data was collected from 50 patients with an average age of 24.5 years, comprising 40% males and 60% females. Among them, 70% had a thin gingival biotype, and 30% had a thick biotype. The mean ANB angle was similar across biotypes (5.5° overall), indicating comparable malocclusion severity. Baseline gingival health metrics showed slight differences between biotypes: thin biotype patients had lower mean gingival thickness (0.88 mm) and width (3.2 mm) than thick biotype patients (1.25 mm and 3.5 mm, respectively). Probing depth and gingival recession were slightly higher in thin biotypes, suggesting a more delicate gingival architecture in this group. The average age was comparable between genders (24.7 years for males and 24.4 years for females). Both groups also had similar ANB angles (5.6° for males and 5.4° for females), plaque index scores (0.8 ± 0.2), and gingival thickness (1.05 mm for males and 0.95 mm for females).

In the thin biotype group, gingival thickness decreased by 0.06 mm, probing depth increased by 0.2 mm, gingival width reduced by 0.2 mm, and gingival recession rose by 0.2 mm. For the thick biotype, changes were less pronounced, with a decrease in gingival thickness by 0.03 mm, a slight increase in probing depth by 0.1 mm, a reduction in gingival width by 0.1 mm, and an increase in gingival recession by 0.1 mm. All changes in the thin biotype group were statistically significant (p < 0.05), highlighting greater susceptibility to gingival alterations following retraction in thin biotype patients compared to those with a thick biotype.

In the thin biotype group, gingival recession rose from 0.5 mm to 0.7 mm, with 28.6% of participants showing an increase, which was statistically significant (p = 0.01). In contrast, the thick biotype group had a minor increase in gingival recession from 0.4 mm to 0.5 mm, with only 13.3% of participants affected, and this change was not statistically significant (p = 0.46). These findings indicate that patients with a thin gingival biotype are more prone to gingival recession following maxillary incisor retraction.

## DISCUSSION

The sequential retraction of maxillary incisors in Orthodontic treatment can have varying effects on the gingival tissue, particularly in patients with different gingival biotypes. This study aimed to assess the impact of maxillary incisor retraction on the maxillary anterior gingiva and found that thin gingival biotypes are more susceptible to negative changes in gingival thickness, probing depth, width, and recession compared to thick biotypes. Mean age of patients in this study was 24.5±3 years and gingival thickness varied from 1.0-1.05 mm in males and females. Other studies measured gingival thickness in age ranges of 29 years which were about 1.5-2mm.^13^

In our study ANB values showing sagittal relationship of maxilla and mandible were taken which did not differ in thin and thick biotypes but other studies have shown that subjects with a smaller ANB value (ANB< 2) had thinner supporting tissue and a keratinized gingival width in the anterior region.^14^ In another study Angles Classification of malocclusion was taken as a criteria and class-1 Div 2 subjects showed more gingival recession due to proclination of teeth.^15^ Gingival biotype plays a crucial role in predicting periodontal outcomes following orthodontic treatment.^8^ The results of our study indicated a statistically significant reduction in gingival thickness and width in patients with a thin biotype.

**Table-III:** Post-Retraction Gingival Changes by Gingival Biotype.

Gingival Parameter	Thin Biotype (n = 35)	Thick Biotype (n = 15)	Mean Change (Thin)	Mean Change (Thick)	p-value
Gingival Thickness (mm)	0.82 ± 0.15	1.22 ± 0.18	-0.06	-0.03	0.03
Probing Depth (mm)	2.3 ± 0.4	2.1 ± 0.3	+0.2	+0.1	0.04
Gingival Width (mm)	3.0 ± 0.5	3.4 ± 0.4	-0.2	-0.1	0.02
Gingival Recession	0.7	0.5	+0.2	+0.1	0.01

**Table-IV:** Gingival Recession Before and After Retraction.

Gingival Biotype	Gingival Recession (mm) Before Retraction	Gingival Recession (mm) After Retraction	Percentage of Participants with Increase	p-value
Thin	0.5	0.7	28.6%	0.01
Thick	0.4	0.5	13.3%	0.46

This reduction can be attributed to the minimal amount of keratinized tissue and thin labial bone present in these patients, making them more vulnerable to gingival recession and attachment loss under orthodontic forces. Similar findings have been reported in previous studies, where thin biotypes showed increased susceptibility to gingival recession during incisor retraction due to less resistance from the gingival and bony structures.^16^,^17^ In the thin biotype group, gingival recession rose from 0.5 mm to 0.7 mm, with 28.6% of participants showing an increase, which was statistically significant (p = 0.01).The retraction of maxillary incisors is a common procedure, particularly in cases where a protrusive dental profile which needs to be managed or in the treatment of Class-II malocclusions. The decrease in gingival thickness and width underscores the need for cautious application of force and possibly modified retraction protocols for patients with thin biotypes to preserve periodontal health.^18^

For patients with thick gingival biotypes, changes in gingival parameters were minimal and not statistically significant, suggesting greater resilience to orthodontic forces. The robust gingival tissue and denser alveolar bone support in thick biotypes provide better protection against recession and attachment loss. This aligns with prior research that thick biotypes generally exhibit more stable periodontal outcomes post-orthodontic treatment.^19^ Thus, in patients with thick gingival biotypes, standard retraction protocols may be applied with less risk of adverse periodontal outcomes. The observed increase in probing depth and gingival recession in the thin biotype group is of particular concern, as these factors contribute to an increased risk of periodontal complications, such as pocket formation and bone loss.^20^ The increase in probing depth could also be a response to stress on the periodontal ligament during tooth movement, which may result in inflammation and soft tissue changes.^21^ Orthodontists should consider these risks when planning treatments for patients with thin gingival biotypes and may benefit from preemptive measures such as reduced force application, slower rates of tooth movement, or incorporating periodontal therapies, like soft tissue grafting, to minimize recession risk.^22^

### Strength:

This study’s findings underscore the importance of identifying gingival biotypes before orthodontic treatment to tailor retraction methods accordingly. A more conservative and personalized approach for patients with thin biotypes could include adjusting force levels, extending retraction times, and monitoring periodontal health closely. Collaborative treatment planning between orthodontists and periodontists may further improve outcomes for patients at higher risk of gingival changes. The strength of this study was that it was done to determine the effects of sequential retraction of maxillary incisors on maxillary anterior gingiva in different gingival biotypes by measuring gingival height, width of keratinized gingiva and probing depth using WHO periodontal probe as there is no such previous study related to the effects of sequential retraction on these gingival parameters.

### Limitations:

The primary limitation of this study is the cross-sectional design, which captures data at a single point in time post-retraction, limiting the understanding of long-term periodontal impacts. Additionally, using non-probability sampling may have introduced selection bias, which could affect the generalizability of the findings. Longitudinal studies with randomized samples could provide more comprehensive insights into the progressive effects of incisor retraction on gingival health. Furthermore, integrating digital imaging and 3D scanning to measure gingival changes over time could enhance the accuracy and depth of future research.

## CONCLUSION

It is concluded that the sequential retraction of maxillary incisors impacts the gingival tissue differently across gingival biotypes, with thin biotypes showing greater susceptibility to changes such as increased probing depth, gingival recession, and reduced gingival thickness. Tailored orthodontic approaches that consider gingival biotypes are essential to minimize periodontal risks, particularly in patients with thin gingival biotypes.

### Recommendations:

Future studies could explore the impact of different retraction techniques and speeds on gingival health across biotypes to develop optimized protocols for managing diverse patient profiles.

### Authors Contribution:

**MIM:** Conceived, designed, did statistical analysis & editing of manuscript, is responsible for integrity of research.

**MIM and SS:** Literature search, Did data collection and manuscript writing.

**AAE and HS:** Literature search, Did review and final approval of manuscript.
